# Legg-Calvé-Perthes Disease Following Nephrotic Syndrome With Long-Term Steroid Use in a Three-Year-Old Boy: A Case Report

**DOI:** 10.7759/cureus.38808

**Published:** 2023-05-09

**Authors:** Manami Ueshima, Atsushi Shimasaki, Tadateru Yasu

**Affiliations:** 1 Department of Pediatrics, National Hospital Organization Nagasaki Medical Center, Ōmura, JPN; 2 Department of Pediatrics, Kameda Medical Center, Kamogawa, JPN; 3 Department of Pediatrics, Nagasaki University Hospital, Nagasaki, JPN

**Keywords:** steroid, nephrotic syndrome, pediatric femoral head necrosis, lcpd, legg-calvé-perthes disease

## Abstract

Nephrotic syndrome (NS) is one of the common pediatric diseases that require glucocorticoid treatment. Patients with NS might receive steroids for a long time if remission is not achieved. Evidence shows that long-term steroid use may induce osteoporosis in adults and children, and steroid use is well known to be related to avascular necrosis of the femoral head (ANFH) in adults. However, no pediatric case of AFNH caused by long-term steroid use due to NS has been reported. In this report, we describe the case of a three-year-old boy with a chief complaint of gait difficulty, who had been treated with glucocorticoid orally for a year because of NS. His body temperature was within the normal limit. His legs did not show trauma, redness, or swelling; however, he did not want his left thigh touched. A pelvic X-ray scan showed asymmetrical femoral heads due to the thinning of the left femoral head. Pelvic magnetic resonance imaging showed a low intensity of the left femoral head on the T2-weighted image and high and low mixed intensities on the fat-suppressed T2-weighted image. Deformation of the left femoral head was suspected. The epiphysial nucleus of the right femoral head was also small for his age. He was diagnosed with Legg-Calvé-Perthes disease and referred to an orthopedic clinic to begin rehabilitation with equipment to support his joints. Thus, we cannot completely conclude that glucocorticoid use and NS are not related to AFNH in children. Physicians must consider early diagnosis.

## Introduction

Nephrotic syndrome (NS) is a common disease in pediatrics, and the standard therapy is glucocorticoid treatment [1]. Glucocorticoid use was often reported to induce avascular necrosis of the femoral head (ANFH) in adults [2]. However, there is no clear evidence regarding the relationship between glucocorticoid use and ANFH in young children without underlying conditions, such as inflammatory, metabolic, hematologic, and bone disorders including fracture histories [3]. Legg-Calvé-Perthes disease (LCPD) is an idiopathic ANFH in children, and its underlying cause is unknown [3]. Lameness and Trendelenburg gait are common complaints during hospital visits, especially in young children, and pain is not always present [3]. Therefore, its diagnosis can be delayed. The incidence of LCPD can vary from 0.4 to 29.0 children per 100,000 children [3]. Although LCPD is an uncommon disease of hip joints in children, its early diagnosis and treatment are essential. Severe cases cause poor prognoses and affect lives tremendously. Children will have high risks for hip osteoarthritis development in the future [4]. Here, we present a case of LCPD following long-term glucocorticoid use in a boy with NS. ANFH after steroid use rarely occurs in children compared with adults [5]. 

## Case presentation

A three-year-old Japanese boy with a history of NS was brought to our hospital with a chief complaint of difficulty in walking since the morning. He was diagnosed with idiopathic NS one year before the presentation. Although the amount of glucocorticoid intake per day had decreased slowly, he had been on oral prednisolone (PDN) treatment for a year continuously without achieving complete remission. Thus far, he had taken 3480 mg of PDN, which was 230 mg/kg of body weight. The maximum amount of PDN per day was 30 mg, which was 2 mg/kg of body weight. It was considered to start using immunosuppressants. However, the patient needed to be referred to the university hospital in the state for that, and it was too far from the family's house. They preferred continuing medications in the community hospital even after getting an explanation of his situation and treatment choices.

On the day of the presentation, he did not want his left upper thigh touched at home, and he had been crying incessantly since the morning. No relevant family history was noted. His birth history, including neonatal metabolic screening, was unremarkable, and his development had been normal. He did not have a cold or a fever. He had no recent trauma or operation history. Vaccinations were up to date. His family history was unremarkable. 

On arrival at the hospital, his vital signs were stable, without fever. His height and weight were within normal limits. On physical examination, no redness or swelling of his left upper thigh was observed; however, he refused to stand up and walk. Left thigh and hip joint pains on palpation were not evident; however, he tried not to bend his left hip joint. Complete blood count and basic metabolic panel were unremarkable. Additional laboratory tests revealed normal total protein and albumin levels. A urine test revealed an elevated protein level at 200 mg/dL (normally negative) without hematuria and bacteriuria, and the urine protein/creatinine ratio was 0.73 g/gCre (normally ≤0.15 g/gCre, ≥2.0 g/gCre in NS). These results show that NS was not recurrent but did not achieve complete remission. C-reactive protein levels were within normal limits.

**Figure 1:**
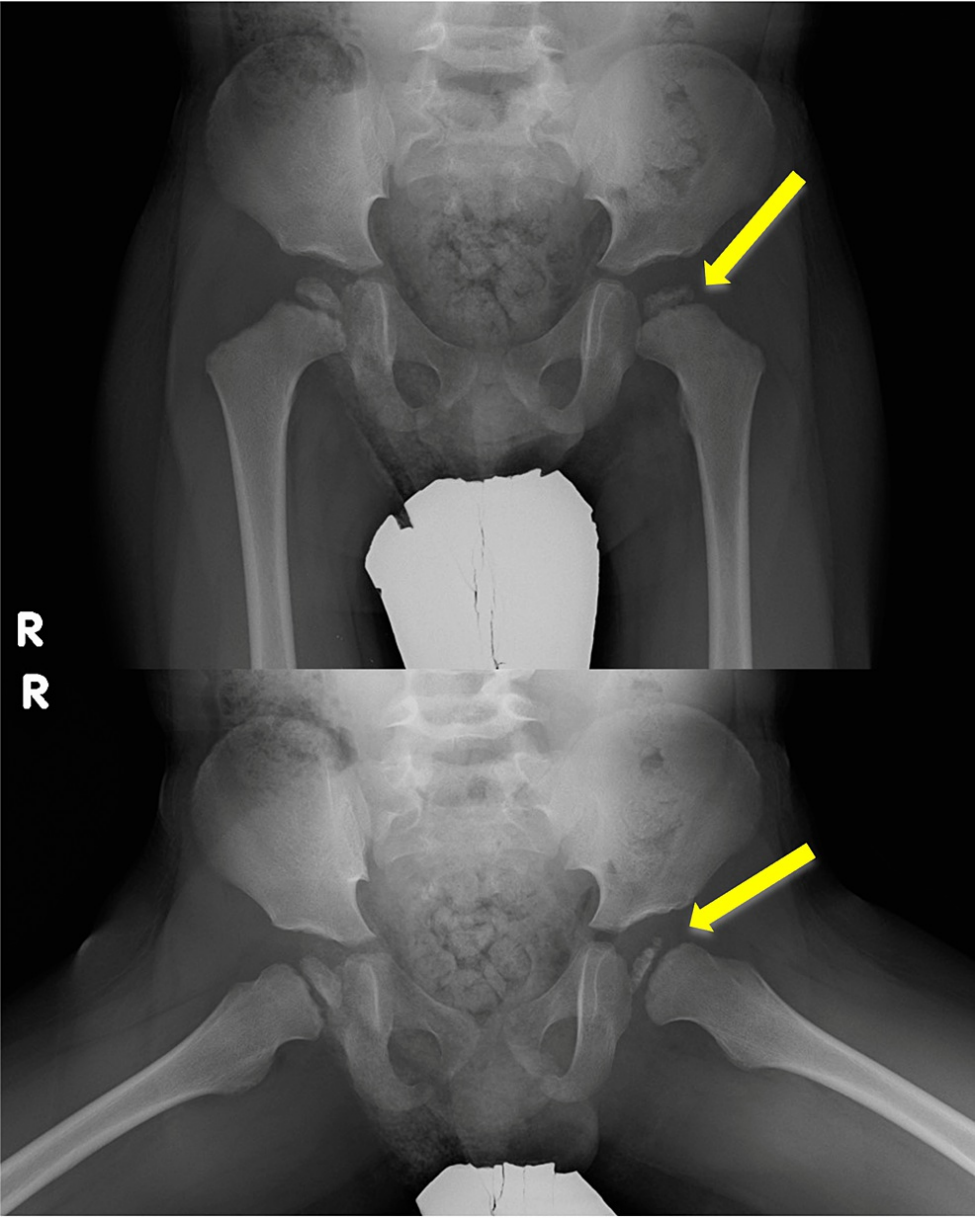
Pelvic X-ray scan showing thinning of the left femoral head (yellow arrows).

**Figure 2:**
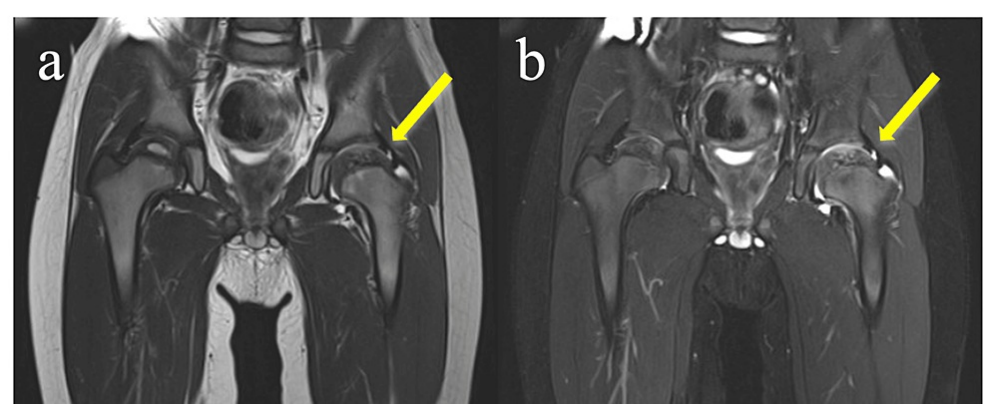
(a) T2-weighted magnetic resonance image of the pelvis showing low intensity of the left femoral head (yellow arrow). (b) Fat-suppressed T2-weighted magnetic resonance image showing high and low mixed intensities of the left femoral head. A little synovial fluid is noticeable (yellow arrow).

A pelvic X-ray scan showed asymmetrical femoral heads and thinning of the left femoral head (Figure 1). Pelvic magnetic resonance imaging revealed low intensity in the left femoral head on the T2-weighted image and high and low mixed intensities in the left femoral head on the fat-suppressed T2-weighted image. Thus, deformation of the left femoral head was suspected, and a small amount of synovial fluid was present (Figure 2). The epiphysial nucleus of the right femoral head was also small for his age. He was diagnosed with LCPD with Catterall grade IV and Herring group C, which indicated a poor prognosis [4]. He was educated not to put his weight on his hips for a while, and he was then referred to an orthopedic clinic to begin rehabilitation with equipment to support his joints [6]. According to the disease classification, he may need surgery in the future [4].

## Discussion

Chronic use of glucocorticoids for NS is not rare in children because of recurrent relapse of the disease [1]. Usually, we need to care about osteoporosis, not femoral head necrosis [4]. Studies in children are limited; however, long-term steroid therapy may increase the risk of fractures in children. In particular, a study reported that pediatric patients receiving glucocorticoid therapy for >3 months should be cared for [7]. In this case, the patient had received steroids for a year, and he had a higher risk of fractures than other children who had not received steroids. 

However, the relationship between ANFH and long-term or high steroid doses per day or the total amount of steroid doses in children has not yet been concluded [5]. As far as we know, no pediatric case of ANFH after receiving steroids due to NS has been reported. Therefore, this is the first case report of LCPD in a pediatric patient receiving steroids for NS. LCPD is a pediatric disease. Although its causes remain unknown, several theories propose environmental, metabolic, and genetic factors [3]. Thus far, steroid use was not reported to cause LCPD.

A study reported a case of recurrent-relapsing NS after LCPD development was detected; however, the LCPD condition did not worsen after long-term use of steroids. That study concluded that steroids do not have adverse effects that can worsen LCPD [8]. However, in the present case, LCPD developed after long-term glucocorticoid use for NS. In addition, throughout his life, the patient has taken over 3000 mg of steroids, which is substantial. Thus, in cases such as this when the cause of ANFH cannot be confirmed, it can be related to steroid use. If so, we need to consider providing immunosuppressant-based treatment to reduce the amount of steroid use in recurrent-relapsing NS more than ever before.

It is also possible that NS can cause ANFH because intravascular dehydration frequently occurs upon loss of albumin in the blood vessels due to NS [9]. Although there is no evidence of the relationship yet, we should consider its possibility. 

In this case, the physician could not detect the LCPD until the patient started experiencing symptoms, and the patient might have a bad prognosis according to the disease classification. LCPD is difficult to diagnose because it can be asymptomatic in the early stage; therefore, routine pelvic X-ray imaging is recommended for follow-up on patients’ hip joints, especially if the relationship between ANFH and steroids or NS in children is established in the future. In addition, careful examination by a pediatrician is crucial.

## Conclusions

We reported a rare case of ANFH in a child after long-term glucocorticoid therapy for NS. LCPD is challenging to diagnose, especially at a young age because early common symptoms are lameness and Trendelenburg gait, not pain. Early diagnosis is essential for a better prognosis of LCPD, and physicians must consider the possibility of this disease. Thus, diagnostic imaging should not be neglected. After experiencing this case, we cannot totally conclude that glucocorticoid therapy and NS are not related to ANFH in children. More case reports and further research are needed.
